# Factors associated with HIV/syphilis co-infection initiating of antiretroviral therapy

**DOI:** 10.11606/s1518-8787.2022056003904

**Published:** 2022-06-20

**Authors:** Luana Andrade Simões, Jullye Campos Mendes, Micheline Rosa Silveira, André Moura Gomes da Costa, Mariana Dias Lula, Maria das Graças Braga Ceccato

**Affiliations:** I Universidade Federal de Minas Gerais Faculdade de Farmácia Programa de Pós-Graduação em Medicamentos e Assistência Farmacêutica Belo Horizonte MG Brasil Universidade Federal de Minas Gerais. Faculdade de Farmácia. Programa de Pós-Graduação em Medicamentos e Assistência Farmacêutica. Belo Horizonte, MG, Brasil; II Universidade Federal de Minas Gerais Faculdade de Farmácia Departamento de Farmácia Social Belo Horizonte MG Brasil Universidade Federal de Minas Gerais. Faculdade de Farmácia. Departamento de Farmácia Social. Belo Horizonte, MG, Brasil; III Universidade Federal de Minas Gerais Faculdade de Engenharia Programa de Pós-Graduação em Engenharia Elétrica Belo Horizonte MG Brasil Universidade Federal de Minas Gerais. Faculdade de Engenharia. Programa de Pós-Graduação em Engenharia Elétrica. Belo Horizonte, MG, Brasil

**Keywords:** HIV Infections, Syphilis, Coinfection, epidemiology, Risk Factors, Antiretroviral Therapy, Highly Active, Cross-Sectional Studies

## Abstract

**OBJECTIVE:**

To evaluate the prevalence and factors associated with HIV/syphilis co-infection in people initiating antiretroviral therapy in Belo Horizonte, capital of the state of Minas Gerais.

**METHODS:**

A sectional section of a prospective cohort study was carried out with people living with HIV, treatment-naive, initiating antiretroviral therapy, older than 16 years, and in follow-up treatment at specialized HIV/Aids care services in Belo Horizonte. Sociodemographic, behavioral, clinical, laboratory and pharmacological treatment-related data were obtained through interviews, medical records, and information systems for logistical control of antiretroviral medications and laboratory tests. The dependent variable was the first episode of active syphilis, recorded by the physician in clinical records, within 12 months after beginning of the antiretroviral therapy. Factors associated with HIV/syphilis co-infection were assessed using binary multiple logistic regression.

**RESULTS:**

Among the 459 individuals included, a prevalence of 19.5% (n = 90) of sexually transmitted infections (STI) was observed, with syphilis (n = 49) being the most frequent STI in these individuals. The prevalence of HIV/syphilis co-infection was 10.6% (n = 49), and the associated independent factors were alcohol use (OR = 2.30; 95%CI: 1.01–5.26), and having a diagnosis of other sexually transmitted infections (OR = 3.33; 95%CI: 1.24–8.95).

**CONCLUSIONS:**

There was a high prevalence of HIV/syphilis co-infection in people living with HIV initiating antiretroviral therapy in Belo Horizonte. HIV/syphilis co-infection was associated with behavioral and clinical factors, such as alcohol use and diagnosis of other sexually transmitted infections. Prior knowledge about the factors associated with this co-infection may support the decisions of health professionals engaged in the care to people living with HIV, with regard to timely diagnosis, guidance, follow-up and adequate treatment, both for syphilis and HIV.

## INTRODUCTION

Sexually transmitted infections (STI) are considered one of the major public health problems in Brazil and worldwide. Their prevention and control entail individual and public benefits, including the decrease in risks of transmission of the human immunodeficiency virus (HIV)^1^.

STIs reach a high rate among sexually active people, and occur silently thus contributing to their dissemination. The syphilis, caused by the etiologic agent *Treponema pallidum*, and HIV infection^2,3^ have the most common transmission routes, and social determinants.

*T. pallidum* infection can increase the viral load and decrease the number of TCD4+ lymphocytes, resulting in increased morbidity and mortality in people living with HIV. Moreover, the presence of HIV may affect the transmission of syphilis, its clinical course, response to treatment, and may change its diagnosis^4^.

Some Brazilian studies found that factors associated with HIV/syphilis co-infection were age, marital status, male gender, low education, multiple partners, presence of STIs, irregular use of condoms, men who have sex with men (MSM), among others^5^. In international studies, the associated factors were male gender, migrants, low education, age, multiple partners, irregular condom use, MSM, illicit drug use, presence of STIs, among others^10^.

This study aimed to assess the prevalence of HIV/syphilis co-infection in HIV-positive individuals at the beginning of antiretroviral therapy (ART) in Belo Horizonte, Minas Gerais, Brazil, and to identify factors associated with HIV/syphilis co-infection.

## METHODS

We carried out a cross-sectional study of a prospective cohort, called Project ECOART “*Efetividade da terapia antirretroviral em pessoas vivendo com HIV/tuberculose, HIV/hanseníase ou HIV/leishmaniose visceral no Brasil*” (Effectiveness of antiretroviral therapy in people living with HIV/tuberculosis, HIV/leprosy, or HIV/visceral leishmaniasis in Brazil). The project was approved by the research ethics committee of the *Universidade Federal de Minas Gerais* (protocol CAAE 31192914.3.3001.5124, opinion CEP 769.085) and of the participating services. Research was conducted in compliance with the instructions of Resolution 466/2012 by the National Health Council.

Sample selection was non-randomized, as all eligible individuals were invited to participate in the study. Recruitment occurred between September 2015 and October 2017.

The study was carried out at three specialized care services (*Serviços de Assistência Especializada*, SAE) in HIV/Aids of the Unified Health System (SUS). SAE I is an outpatient clinic of a state reference hospital for the treatment of infectious diseases and health dermatology; SAE II is a testing and counseling center (*Centro de Testagem e Aconselhamento*, CTA); SAE III is a reference SAE for the care of infectious and parasitic diseases.

The eligibility criteria were: individuals of both genders, aged 16 years or older, diagnosed with HIV regardless of the time of diagnosis and the clinical condition of the individual, starting ART (from zero to six months of treatment), without previous pharmacological treatment of HIV infection, and who were being followed-up at one of the three selected services. All participants agreed to participate in the study and signed the informed consent form.

The dependent variable was the first episode of active syphilis recorded by the physician in the medical record within 12 months after initiation of ART.

The independent variables related to sociodemographic, behavioral, clinical, laboratory, and pharmacological treatment information were obtained through face-to-face interviews, data collection from clinical records, the Logistic Medication Control System (*Sistema de Controle Logístico de Medicamentos,* Siclom), and the National CD4+/CD8+ Lymphocyte Count and HIV Viral Load Network Laboratory Test Control System (*Sistema de Controle de Exames Laboratoriais da Rede Nacional de Contagem de Linfócitos* CD4+/CD8+ *e Carga Viral do HIV*, Siscel).

The age of individuals was stratified into age groups for descriptive analysis, and as a continuous variable in logistic regression. The variable self-reported color or ethnicity was stratified into white, black, yellow, brown, or indigenous. Marital status was dichotomized into single/divorced/widowed or married/common-law marriage. Education was categorized as up to 9 years, 10 to 12 years, and 13 years or more of formal education for descriptive analysis, and in two categories for logistic regression (up to 9 years, 10 years or more). We also investigated the existence of children, whether or not they have a job, their own income, private health insurance, place of residence, and economic class.

The economic class variable was evaluated according to Brazilian criteria, such as high (A-B), intermediate (C) and low (D-E). Here, individuals are classified by socioeconomic groups according to possession of comfort items, and the family head’s level of education. For analysis, the variable was categorized into high (A-B) and intermediate-low (C-D-E).

In the evaluation of behavioral variables and lifestyle habits, we analyzed the existence of a fixed sexual partner within 12 months after starting ART, alcohol use in the month before the baseline interview, tobacco use at the time of the interview, use of illicit drugs ever in life (marijuana, cocaine, crack, and others, such as ecstasy and glue sniffing), and condom use in the last month and during the last sexual intercourse.

We analyzed the average time of HIV diagnosis by self-report, the initial and final clinical classification of the individual - categorized as A (asymptomatic), B (symptomatic), and C (Aids-defining clinical condition), according to the criteria of the adapted Centers for Disease Control and Prevention (CDC)^13^ , the presence of comorbidities, presence of other previous and current STIs, presence of mucous lesions (oral, genital, or anal), viral load, and TCD4 lymphocyte count, according to the Siscel data, mean duration of ART, and treatment regimens, according to the Siclom data. Treatment regimens were categorized as tenofovir/lamivudine/efavirenz (TLE), tenofovir/lamivudine/dolutegravir (TLD), and other (any other antiretroviral regimen). Non-adherence was assessed by self-report, using the question “Have you missed the medication in the last 15 days (yes; no)?”

Descriptive analysis was carried out using frequency distribution for categorical variables, and measures of central tendency and variability for continuous variables. Pearson’s chi-square or Fischer’s exact test was used to compare proportions between categorical variables, and the T-test was used to compare means between continuous variables. The multiple binary logistic regression model was used to assess factors associated with HIV/ syphilis co-infection.

All variables were subjected to collinearity tests. The results of logistic regression were presented by odds ratio (OR), 95% confidence interval (95%CI), and p value. Variables that showed p value of 0.20 or less in the bivariate analysis were included in the multivariate model. The stepwise backward conditional method was used to obtain the final model. The Hosmer-Lemeshow test and the area under the Receiver Operating Characteristics (ROC) curve were used to verify the model fit. Statistical analyses were performed using the Statistical Package for the Social Sciences (SPSS) software, version 22.0, and R Studio, version 4.0.2. All analyses were performed with a 5% significance level.

## RESULTS

A total of 459 individuals were enrolled in the study (Figure 1). Among the sociodemographic characteristics of the study general population, it was observed that 81.5% of the individuals were male, with a mean age of 34.7 years (SD = 10.9), and a predominance of the age range of 20 to 34 years (53.4%). Most individuals were single, divorced, or widowed (79.7%), and had 10 years or more of formal education (74.3%). Regarding behavioral characteristics, 27.5% of the individuals used tobacco at the time of the interview, 64.1% used alcohol in any quantity in the month before the interview, 48.1% used illicit drugs at some time in their lives. As for the potential source of HIV infection, more than half (51%) were MSM.

**Figure:**
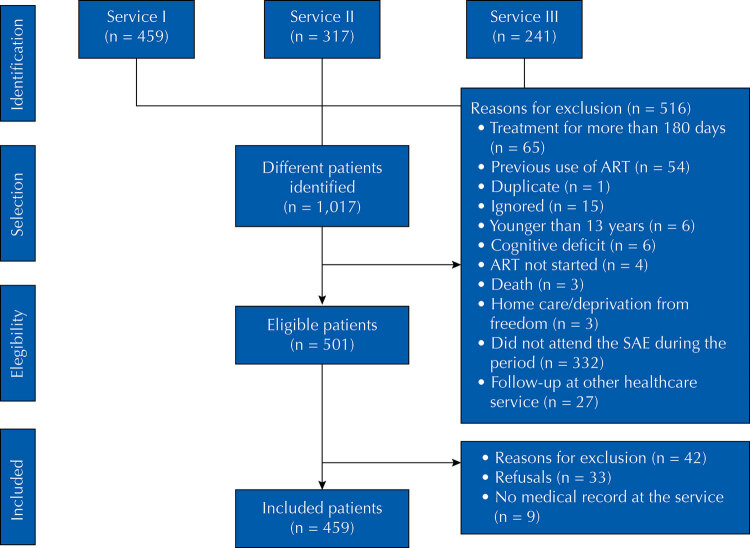
Diagram of the individuals included in the study.

ART: antiretroviral therapy.

Among the participants, 25.5% reported not using condoms in the last month, and 21.4% in the last sexual intercourse. Clinical classification A (asymptomatic) was observed in 63.4% of individuals at the first visit, and in 80.8% at the last visit. At the beginning of treatment, most participants (88.9%) had detectable viral load and, at the end of 12 months of ART, only 6.8% had detectable viral load. The percentage of missing data was 30.5%. At the beginning of ART, we observed that 26.1% of the individuals had TCD4 lymphocyte counts below 200 cells/mm^3^, and 20.9% started treatment with clinical conditions indicative of Aids. Regarding treatment-related characteristics, the mean time to ART initiation was 78.5 days (SD = 58.97); 63.4% of individuals were using the TDF/3TC/EFV regimen; 14.8% reported non-adherence to treatment; and, 70.8% had been diagnosed with HIV for more than three months (Table 1).

**Table 1:** Sociodemographic, clinical, behavioral characteristics, life habits, and treatment of PLHIV, Belo Horizonte, Minas Gerais, 2015–2018 (n = 459).

Characteristics	Total n = 459		HIV/ syphilis co-infection				p
			Yes n = 49		No n = 410		
	n	%	n	%	n	%	
**Sociodemographic**							
Sex							0.018^a^
Male	374	81.5	46	93.9	328	80.0	
Female	85	18.5	3	6.1	82	20.0	
Age (years)							0.573
16–19	18	3.9	1	2.0	17	4.1	
20–34	245	53.4	26	53.1	219	53.4	
35–49	145	31.6	14	28.6	131	32.0	
≥ 50	51	11.1	8	16.3	43	10.5	
Color/ethnicity							0.874^a^
Brown	222	48.4	21	42.9	201	49.0	
White	108	23.5	13	26.5	95	23.2	
Black	106	23.1	14	28.6	92	22.4	
Yellow	15	3.3	1	2.0	14	3.4	
Indigenous	3	0.7	0	0.0	3	0.7	
Missing data	5	1.1	0	0.0	5	1.2	
Marital status							0.271
Single/divorced/widowed	366	79.7	42	85.7	324	79.0	
Married/commonwealth marriage	93	20.3	7	14.3	86	21.0	
Children							0.297
Yes	162	35.3	14	28.6	148	36.1	
No	297	64.7	35	71.4	262	63.9	
Formal education (years)							0.043^a^
≤ 9	117	25.5	11	22.4	106	25.9	
10–12	178	38.8	12	24.5	166	40.5	
≥ 13	163	35.5	26	53.1	137	33.4	
Missing data	1	0.2	0	0.0	1	0.2	
Job							0.694
Yes	269	58.6	30	61.2	239	58.3	
No	190	41.4	19	38.8	171	41.7	
Own income							0.087^a^
Yes	373	81.3	41	83.7	332	81.0	
No	85	18.5	7	14.3	78	19.0	
Missing data	1	0.2	1	2.0	0	0.0	
Economic class							0.306^a^
High (A-B)	162	35.3	20	40.8	142	34.6	
Intermediate (C)	212	46.2	23	46.9	189	46.1	
Low (D-E)	73	15.9	4	8.2	69	16.8	
Missing data	12	2.6	2	4.1	10	2.4	
Private health plan							0.511
Yes	121	26.4	11	22.4	110	26.8	
No	338	73.6	38	77.6	300	73.2	
Place of residence							0.656^a^
Belo Horizonte	404	88.0	44	89.8	360	87.8	
Belo Horizonte Metropolitan Area	49	10.7	4	8.2	45	11.0	
Other municipalities	6	1.3	1	2.0	5	1.2	
**Behavioral and life habits**							
Fixed sexual partner within 12 months after ART							0.619^a^
Yes	218	47.5	22	44.9	196	47.8	
No	187	40.7	23	46.9	164	40.0	
Missing data	54	11.8	4	8.2	50	12.2	
Use of alcohol in the month before the interview							0.435^a^
Yes	294	64.1	36	73.5	258	62.9	
No	162	35.3	13	26.5	149	36.3	
Missing data	3	0.7	0	0.0	3	0.7	
Current use of tobacco							0.600
Yes	126	27.5	15	30.6	111	27.1	
No	333	72.5	34	69.4	299	72.9	
Use of illicit drugs in life ever							0.493^a^
Yes	221	48.1	27	55.1	194	47.3	
No	236	51.4	22	44.9	214	52.2	
Missing data	2	0.4	0	0.0	2	0.5	
Condom use in the last month							0.156
Yes	248	54.0	31	63.3	217	52.9	
No	117	25.5	13	26.5	104	25.4	
Missing data	94	20.5	5	10.2	89	21.7	
Condom use in the last sexual intercourse							0.383^a^
Yes	343	74.7	41	83.7	302	73.7	
No	98	21.4	7	14.3	91	22.2	
Missing data	18	3.9	1	2.0	17	4.1	
Source of HIV infection exposure category							0.025^a^
Men (MSM)	234	51.0	34	69.4	200	48.8	
Men (non MSM)/women	168	36.6	12	24.5	156	38.0	
Missing data	57	12.4	3	6.1	54	13.2	
**Clinical**							
Clinical classification at the first visit							0.907^a^
Aids conditions (C)	96	20.9	11	22.4	85	20.7	
Asymptomatic (A)	291	63.4	30	61.2	261	63.7	
Symptomatic (B)	67	14.6	8	16.3	59	14.4	
Missing data	5	1.1	0	0.0	5	1.2	
Clinical classification at the last visit							0.351^a^
Aids conditions (C)	35	7.6	5	10.2	30	7.3	
Asymptomatic (A)	371	80.8	42	85.7	329	80.2	
Symptomatic (B)	16	3.5	0	0.0	16	3.9	
Missing data	37	8.1	2	4.1	35	8.5	
Comorbidities							0.951^a^
Yes	172	37.5	18	36.7	154	37.6	
No	279	60.8	31	63.3	248	60.5	
Missing data	8	1.7	0	0.0	8	2.0	
Other STIs (except syphilis)							0.021
Yes	41	8.9	9	18.4	32	7.8	
No	418	91.1	40	81.6	378	92.2	
Mucosal lesions							0.086^a^
Yes	23	5.0	5	10.2	18	4.4	
No	436	95.0	44	89.8	392	95.6	
History of STI							0.204
Yes	134	29.2	16	32.7	118	28.8	
No	124	27.0	9	18.4	115	28.0	
Missing data	201	43.8	24	49.0	177	43.2	
**Laboratory**							
Viral load at start of ART (copies/ml)							0.812^a^
≤ 100 thousand	305	66.4	31	63.3	274	66.8	
> 100 thousand	114	24.8	14	28.6	100	24.4	
Missing data	40	8.7	4	8.2	36	8.8	
Viral load at start of ART							0.194^a^
Undetectable	11	2.4	3	6.1	8	2.0	
Detectable	408	88.9	42	85.7	366	89.3	
Missing data	40	8.7	4	8.2	36	8.8	
Viral load after 12 months of ART (copies/ml)							0.211^a^
≤ 100 thousand	316	68.8	39	79.6	277	67.6	
> 100 thousand	3	0.7	0	0.0	3	0.7	
Missing data	140	30.5	10	20.4	130	31.7	
Viral load after 12 months of ART							0.203
Undetectable	288	62.7	34	69.4	254	62.0	
Detectable	31	6.8	5	10.2	26	6.3	
Missing data	140	30.5	10	20.4	130	31.7	
TCD4 lymphocytes at start of ART (cells/mm^3^)							0.274^a^
< 200	120	26.1	12	24.5	108	26.3	
200–500	165	35.9	13	26.5	152	37.1	
> 500	134	29.2	20	40.8	114	27.8	
Missing data	40	8.7	4	8.2	36	8.8	
TCD4 lymphocytes after 12 months of ART (cells/mm^3^)							0.998
< 200	27	5.9	3	6.1	24	5.9	
200–500	139	30.3	15	30.6	124	30.2	
> 500	155	33.8	16	32.7	139	33.9	
Missing data	138	30.1	15	30.6	123	30.0	
**Drug treatment**							
Mean time of HIV diagnosis (month)	15.3	SD = 32.1	8.5	SD = 13.4	16.1	SD = 33.5	0.004^b^
Time of HIV diagnosis (month)							0.018^a^
≤ 3	131	28.5	21	42.9	110	26.8	
> 3	325	70.8	27	55.1	298	72.7	
Missing data	3	0.7	1	2.0	2	0.5	
Average time of ART treatment (days)	78.6	SD = 59.0	62.8	SD = 55.2	80.4	SD = 59.2	0.048^b^
Therapeutic regimen in use							0.085^a^
TLE	291	63.4	38	77.6	253	61.7	
TLD	142	30.9	9	18.4	133	32.4	
Other regimens	26	5.7	2	4.1	24	5.9	
Non-adherence to ART in the last 15 days							0.339
Yes	68	14.8	9	18.4	59	14.4	
No	362	78.9	35	71.4	327	79.8	
Missing data	29	6.3	5	10.2	24	5.9	

PLHIV: people living with HIV; MSM: men who have sex with men; STI: sexually transmitted infections; TLE: tenofovir/lamivudine/efavirenz; TLD: tenofovir/lamivudine/dolutegravir; ART: antiretroviral therapy; SD: standard deviation.

^a^ Fischer’s exact test.

^b^ T-test for comparison of means.

We observed a 19.6% (n = 90) prevalence of STIs registered on the medical record, with more than half of the records being syphilis (n = 49). Among the 41 individuals diagnosed with STIs other than syphilis, we found 9.4% (n = 20) of condyloma and genital warts (anal, vaginal, perianal); 5.6% (n = 12) of genital herpes; 5.6% (n = 12) of hepatitis B and C; and 1.4% (n = 3) of other STIs (gonorrhea/trichomoniasis/unspecified STI). Other oral, anal or genital mucous lesions were also recorded (5%), described as penile lesion, erythematous itchy penis lesion, anal lesion, and genital lesion (data not shown in table).

The prevalence of HIV/syphilis co-infected individuals in this study was 10.6% (n = 49). As for the clinical characteristics of these co-infected individuals, it was observed that most had unspecified syphilis (45%), followed by latent and late latent syphilis (26.6%), secondary (14.3%), tertiary (neurosyphilis and uveitis) (12.1%), and primary (primary genital) syphilis (2%). The mean time of the first syphilis episode after initiation of ART was 115.06 days (SD = 121.26), and the median was 53 days. The test reported in the registry was Venereal Disease Research Laboratory (VDRL), with 42.9% reagent results, and 55.1% had no test record. Penicillin G Benzathine was prescribed for 85.7% of the individuals being treated for syphilis, and 4.1% used other drugs such as doxycycline and ceftriaxone. We also observed 18.4% (n = 9) of other STIs, additionally to the co-infection, being these condyloma and genital warts, hepatitis B and C, and others (gonorrhea/trichomoniasis/unspecified STIs). It was also observed that 10.2% had some record of lesions in oral, anal or genital mucosa (data not shown in the table).

As shown in Table 1, most co-infected individuals were male (93.9%), aged 20 to 34 years (53.1%), brown or black (71.5%), divorced/single/widowed (85.7%). As for behavioral characteristics, most were MSM (69.4%), had no fixed sexual partner (46.9%), and used alcohol in the month before the baseline interview (73.5%). As for condom use, 26.5% reported not having used in the last month, and 14.3% did not use in the last sexual intercourse. There were differences between the groups with and without co-infection for the variables sex (p = 0.018), formal education (p = 0.043), source of HIV infection (p = 0.025), diagnosis of other STIs (p = 0.021), mean time of HIV diagnosis (p = 0.004), and mean time of antiretroviral treatment (p = 0.048) (Table 1).

The characteristics significantly associated with a higher chance of having HIV/syphilis co-infection in the bivariate analysis were male sex, being MSM, and having a diagnosis of other STIs. The characteristics associated with a lower chance of HIV/syphilis co-infection were longer duration of ART, and use of the TLD antiretroviral regimen (Table 2).

**Table 2:** Bivariate analysis of factors associated with HIV/syphilis coinfection, Belo Horizonte, Minas Gerais, 2015–2018 (n = 459).

Characteristics	n (%)^a^	OR (95%CI)	p
**Sociodemographic**			
Sex			
Male	374 (81.5)	3.83 (1.16–12.64)	0.027
Female	85 (18.5)	1.00	
Age (years)	459 (100)	1.01 (0.98–1.03)	0.674
Color/ethnicity			
Brown/black	328 (72.2)	0.96 (0.50–1.84)	0.892
White/yellow/indigenous	126 (27.8)	1.00	
Marital status			
Single/divorced/widowed	366 (79.7)	1.59 (0.69–3.67)	0.275
Married/commonwealth marriage	93 (20.3)	1.00	
Children			
Yes	162 (35.3)	1.00	
No	297 (64.7)	1.41 (0.74–2.71)	0.299
Formal education (years)			
≤ 9	117 (25.5)	0.83 (0.41–1.68)	0.599
≥ 10	341 (74.5)	1.00	
Job			
Yes	269 (58.6)	1.00	
No	190 (41.4)	0.89 (0.48–1.63)	0.694
Own income			
Yes	373 (81.4)	1.00	
No	85 (18.6)	0.73 (0.31–1.68)	0.456
Economic class			
High (A-B)	162 (36.2)	1.00	
Intermediate-low (C-D-E)	285 (63.8)	0.74 (0.40–1.37)	0.343
Private health plan			
Yes	121 (26.4)	1.00	
No	338 (73.6)	1.27 (0.63–2.57)	0.511
Place of residence			
Metropolitan region/other municipalities	55 (12.0)	1.00	
Belo Horizonte	404 (88.0)	1.22 (0.46–3.23)	0.685
**Behavioral and life habits**			
Fixed sexual partner within 12 months after ART			
Yes	218 (47.5)	1.00	
No	187 (40.7)	1.25 (0.67–2.32)	0.482
Use of alcohol in the month before the interview			
Yes	294 (64.5)	1.60 (0.82–3.11)	0.167
No	162 (35.5)	1.00	
Current use of tobacco			
Yes	126 (27.5)	1.19 (0.62–2.27)	0.600
No	333 (72.5)	1.00	
Use of illicit drugs in life ever			
Yes	221 (48.4)	1.35 (0.75–2.46)	0.319
No	236 (51.6)	1.00	
Condom use in the last month			
Yes	248 (67.9)	1.00	
No	117 (32.1)	0.88 (0.44–1.74)	0.704
Condom use in the last sexual intercourse			
Yes	343 (77.8)	1.00	
No	98 (22.2)	0.57 (0.25–1.31)	0.182
Source of HIV infection - exposure category			
Men (MSM)	234 (51.0)	2.21 (1.11–4.41)	0.024
Men (non MSM)/women	168 (36.6)	1.00	
**Clinical**			
Clinical classification at the first visit			
Aids conditions (C)	96 (21.1)	1.00	
No Aids (A-B)	358 (78.9)	1.09 (0.54–2.22)	0.813
Clinical classification at the last visit			
Aids conditions (C)	35 (8.3)	1.00	
No Aids (A-B)	387 (91.7)	0.73 (0.27–1.98)	0.538
Comorbidities			
Yes	172 (38.1)	0.94 (0.51–1.73)	0.830
No	279 (61.9)	1.00	
Other STIs (except syphilis)			
Yes	41 (8.9)	2.66 (1.19–5.96)	0.018
No	418 (91.1)	1.00	
Mucosal lesions			
Yes	23 (5.0)	2.48 (0.88–6.99)	0.087
No	436 (95.0)	1.00	
**Laboratory**			
Viral load at start of ART			
Detectable	408 (88.9)	1.00	
Undetectable	11 (2.4)	3.27 (0.84–12.79)	0.089
Viral load after 12 months of ART			
Detectable	31 (6.8)	1.00	
Undetectable	288 (62.7)	0.70 (0.25–1.93)	0.487
TCD4 lymphocytes at start of ART (cells/mm^3^)			
< 200	120 (26.1)	1.00	
200–500	165 (35.9)	0.77 (0.34–1.75)	0.533
> 500	134 (29.2)	1.58 (0.74–3.39)	0.240
TCD4 lymphocytes after 12 months of ART (cells/mm^3^)			
< 200	27 (5.9)	1.00	
200–500	139 (30.3)	0.97 (0.26–3.60)	0.961
> 500	155 (33.8)	0.92 (0.25–3.40)	0.902
**Drug treatment**			
Time of HIV diagnosis (months)	456 (100.0)	0.99 (0.97–1.01)	0.138
Average time of antiretroviral treatment (days)	459 (100.0)	0.99 (0.99–1.00)	0.050
Therapeutic regimen in use			
TLE	291 (63.4)	1.00	
TLD	142 (30.9)	0.45 (0.21–0.96)	0.039
Other regimens	26 (5.7)	0.56 (0.13–2.44)	0.436
Self-report of non-adherence to ART in the last 15 days			
Yes	68 (15.8)	1.43 (0.65–3.12)	0.375
No	362 (84.2)	1.00	

OR: odds ratio; 95%CI: 95% confidence interval; ART: antiretroviral therapy; MSM: men who have sex with men; STI: sexually transmitted infections; TLE: Tenofovir/Lamivudine/Efavirenz; TLD: Tenofovir/Lamivudine/Dolutegravir.

^a^ Numbers vary as data are ignored.

In the multivariate analysis (Table 3), the independent characteristics associated with a higher chance of co-infection were having been diagnosed with other STIs (OR = 3.33; 95%CI: 1.24–8.95), and alcohol use in the month before the interview (OR = 2.30; 95%CI: 1.01–5.26). The variables sex and length of antiretroviral treatment remained in the final model, but did not report statistical significance.

**Table 3:** Multiple logistic regression of factors associated with HIV/syphilis co-infection, Belo Horizonte, Minas Gerais, 2015–2018 (n = 349a).

Characteristics	OR (95%CI)	p
Sex		
Male	3.58 (0.82–15.58)	0.089
Female	1.00	
Use of alcohol in the month before the interview		
Yes	2.30 (1.01–5.26)	0.049
No	1.00	
Diagnosis of other STIs (except syphilis)		
Yes	3.33 (1.24–8.95)	0.017
No	1.00	
Average time of antiretroviral treatment (days)	0.99 (0.99–1.00)	0.066

OR: odds ratio; 95%CI: 95% confidence interval. STI: sexually transmitted infections; ROC: Receiver Operating Characteristics.

^a^ 110 patients with missing data in covariates were excluded from the final model.

Model fit: Hosmer and Lemeshow test: X2 = 7.66; df = 8; p-value = 0.468; area under the ROC curve = 0.688.

## DISCUSSION

People living with HIV (PLHIV) seen in three public specialized HIV care services in Belo Horizonte, who were starting ART, showed high prevalence of STI co-infection (19.6%), with syphilis (10.6%) being the most prevalent. It is noteworthy that the estimated overall prevalence of syphilis among men and women without HIV infection is 0.5% in Brazil^1^. Characteristics independently associated with HIV/syphilis co-infection were diagnosis of other STIs, and use of alcohol in the month prior to the interview.

The prevalence and factors associated with syphilis in PLHIV vary both in Brazilian and international studies. This variation depends on the type of population, such as the key population (transgender, sex workers, people who inject drugs, MSM, and prisoners - and their partners) that have higher prevalence of HIV/syphilis co-infection^14^.

The prevalence of HIV/syphilis co-infection observed in this study was lower than that found in a study conducted in Mkushi, Zambia, in which the authors observed 40.5% HIV/syphilis co-infection in newly diagnosed HIV-positive individuals starting ART^15^. It was also lower than the prevalence found in a prospective multicenter study of MSM in Germany, which was 39.6%^16^. In another study conducted in Brazil with sex workers, the prevalence was 30.8%^17^.

Similar to other studies^18,19^, we also found that having been diagnosed with other STIs was independently associated with a higher chance of HIV/syphilis co-infection. This result may indicate that risky sexual behavior among PLHIV may contribute to the spread of HIV infection, and affect the transmission control.

The STIs are transmitted by sexual contact without the use of condoms, an important preventive measure among HIV serodiscordant and seroconcordant couples to prevent the transmission of other STIs. One study found the presence of syphilis, cytomegalovirus, human papillomavirus (HPV), and herpes simplex virus in MSM living with HIV^20^. Similarly, in our study we found the presence of condylomata and genital warts, genital herpes, hepatitis B and C and others (gonorrhea/trichomoniasis/unspecified STDs), besides lesions in anal and genital mucosa.

PLHIV are at higher risk of co-infection with hepatitis and syphilis than the population at large. Bacterial infections, protozoa, genital herpes, and previous sexual infections have been described as risk factors for HIV/syphilis co-infection. STIs may indicate risky sexual behavior among PLHIV, increasing the possibility of HIV infection and affecting the control of transmission^20^_._

Another study reviewed the factors associated with HIV/STI co-infection in 295 PLHIV, in which 37% had at least one STI. Among the STIs cited, 32% were syphilis, 16% gonorrhea, and 8% chlamydia. The high prevalence of STIs among PLHIV suggests the need for adequate testing, prevention, and treatment among this population^21^.

In this study, reports of alcohol use prior to the interview were associated with a higher chance of HIV/syphilis co-infection, a result similar to other studies^22,23^. Alcohol consumption is a serious public health problem since it may lead individuals to adopt risky sexual practices and contribute to the lack of STI preventive habits, such as not using condoms, changing partners frequently, and engaging in group or anal sex, leading to increased chances of contracting syphilis and other STIs. This scenario contributes to maintaining the chain of transmission of STIs among PLHIV^24^_._

Alcohol is a substance that depresses the central nervous system, and reduces anxiety and inhibition. The belief that using alcohol increases pleasure causes it to be used before or during sexual practices. It is estimated that alcohol consumption among PLHIV is 2.5 times higher than in the remaining population. The use of alcohol and drugs increases up to six times the risk of people with HIV to have unprotected sex and multiple partners. In one study, the prevalence of alcohol abuse among people living with HIV was estimated at 28.6%^25^_._

Longer duration of ART was associated with lower chance of HIV/syphilis co-infection, and remained in the final model due to greater robustness, although it did not show statistical significance. It is noteworthy that the results of studies evaluating the association between ART use and STI transmission are controversial.

A retrospective cohort study found an association between the use of ART and a lower chance of HIV/syphilis co-infection at the beginning of treatment, supporting the results found in this study. On the other hand, ART use was associated with higher chance of co-infection in individuals who had syphilis seroconversion during follow-up^26^. In a study by Tsachouridou et al.^27^, individuals taking ART were 2.4 times more likely to have HIV/syphilis co-infection. These studies indicate that the advantages of antiretroviral use are reflected in the sexual behavior of PLHIV of not using condoms. It is likely to be so because feel safe about not transmitting the HIV virus^27^.

In our study, the male sex variable was associated with lower chance of HIV/syphilis co-infection, and remained in the final model due to greater robustness, although it did not show statistical significance. This result was consistent with that of other studies that showed a higher risk of co-infection among males^28,29^.

The prevalence of syphilis and the different STIs found in this study may reflect the inconsistent use of condoms, and other actions to prevent these infections. Awareness about the factors associated with the prevalence of HIV/syphilis co-infection may support the decision-making of professionals involved in the care of PLHIV. The follow-up and adequate treatment of syphilis and STIs require guidance on safe sex practices to prevent these co-infections among the PLHIV.

The limitations of the study concern the use of secondary data with missing elements of general records on clinical information, and laboratory tests of individuals.

The strengths of this study are the quality and processing of primary data collected, with reliability analysis of 10% of the total sample for collection and entry. Of note is the high perfect interdigitated agreement assessed by Kappa statistics, the comprehensive inclusion of explanatory variables, and the robustness of the final model.

The conclusion is that the prevalence of STIs recorded was high, and syphilis was the most prevalent co-infection. Alcohol use and diagnosis of other STIs were associated with a higher chance of HIV/syphilis co-infection among this population.
