# Supplementary material for: Factors associated with HIV/syphilis co-infection initiating of antiretroviral therapy
Source: Rev Saude Publica. 2022 Jun 20;56:59. doi: 10.11606/s1518-8787.2022056003904 (PMC9239423; doi:10.11606/s1518-8787.2022056003904)
Supplement: Translation

# Translation: Fatores associados à coinfecção HIV/sífilis no início da terapia antirretroviral

**Authors:** Luana Andrade Simões, Jullye Campos Mendes, Micheline Rosa Silveira, André Moura Gomes da Costa, Mariana Dias Lula, Maria das Graças Braga Ceccato

**Affiliations:** I Brasil Universidade Federal de Minas Gerais. Faculdade de Farmácia. Programa de Pós-Graduação em Medicamentos e Assistência Farmacêutica. Belo Horizonte, MG, Brasil; II Brasil Universidade Federal de Minas Gerais. Faculdade de Farmácia. Departamento de Farmácia Social. Belo Horizonte, MG, Brasil; III Brasil Universidade Federal de Minas Gerais. Faculdade de Engenharia. Programa de Pós-Graduação em Engenharia Elétrica. Belo Horizonte, MG, Brasil

**Keywords:** Infecções por HIV, Sífilis, Coinfecção, epidemiologia, Fatores de Risco, Terapia Antirretroviral de Alta Atividade, Estudos Transversais

## Abstract

**OBJETIVO:**

Avaliar a prevalência e os fatores associados à coinfecção HIV/sífilis em pessoas no início da terapia antirretroviral no município de Belo Horizonte, Minas Gerais.

**MÉTODOS:**

Foi realizado um corte seccional de um estudo de coorte prospectivo, com pessoas vivendo com HIV, sem tratamento prévio da infecção, em início da terapia antirretroviral, maiores de 16 anos e em acompanhamento em serviços de assistência especializada em HIV/aids de Belo Horizonte. Dados sociodemográficos, comportamentais, clínicos, laboratoriais e relacionados ao tratamento farmacológico foram obtidos por meio de entrevistas, coleta em prontuários clínicos e nos sistemas de informação de controle de medicamentos antirretrovirais e exames laboratoriais. A variável dependente foi o primeiro episódio de sífilis ativa, registrado pelo médico em prontuário clínico, em um período de 12 meses após início da terapia antirretroviral. Os fatores associados à coinfecção HIV/sífilis foram avaliados por meio de regressão logística binária múltipla.

**RESULTADOS:**

Dentre os 459 indivíduos avaliados, observou-se uma prevalência de 19,5% (n = 90) de infecções sexualmente transmissíveis, sendo a sífilis (n = 49) a infecção sexualmente transmissível mais frequente nesses indivíduos. A prevalência da coinfecção HIV/sífilis foi de 10,6% (n = 49) e os fatores independentes associados foram o uso de álcool (OR = 2,30; IC95% 1,01–5,26) e ter diagnóstico de outras infecções sexualmente transmissíveis (OR = 3,33; IC95% 1,24–8,95).

**CONCLUSÕES:**

Houve alta prevalência de coinfecção HIV/sífilis em pessoas vivendo com HIV em início de terapia antirretroviral em Belo Horizonte. A coinfecção HIV/sífilis foi associada a fatores comportamentais e clínicos, como uso de álcool e diagnóstico de outras infecções sexualmente transmissíveis. O conhecimento prévio sobre os fatores associados à essa coinfecção pode subsidiar as decisões dos profissionais de saúde inseridos no cuidado às pessoas vivendo com HIV, no que diz respeito ao diagnóstico oportuno, orientações, acompanhamento e tratamento adequado, tanto da sífilis quanto do HIV.

## INTRODUÇÃO

As infecções sexualmente transmissíveis (IST) são consideradas um dos maiores problemas de saúde pública no Brasil e no mundo, e sua prevenção e controle têm benefícios individuais e públicos, dentre eles a diminuição dos riscos de transmissão do vírus da imunodeficiência humana adquirida (HIV)^1^.

As IST atingem um alto índice entre as pessoas sexualmente ativas e ocorrem de forma silenciosa, o que contribui para a sua disseminação e as que possuem modo de transmissão e determinantes sociais mais comuns são a sífilis, causada pelo agente etiológico *Treponema Pallidum,* e a infecção pelo HIV^2,3^.

A infecção pelo *T. pallidum* pode aumentar a carga viral e diminuir o número de linfócitos TCD4+, resultando em uma condição maior de morbidade e mortalidade na pessoa que vive com HIV. Além disso, a presença do HIV pode afetar a transmissão da sífilis, seu curso clínico, resposta ao tratamento e alterar seu diagnóstico^4^.

Em alguns estudos brasileiros, os fatores associados à coinfecção HIV/sífilis foram idade, estado civil, sexo masculino, baixa escolaridade, multiplicidade de parceiros, presença de IST, uso irregular de preservativos, homens que fazem sexo com homens (HSH), dentre outros^5^. Em estudos internacionais, os fatores associados foram sexo masculino, migrantes, baixa escolaridade, idade, múltiplos parceiros, uso irregular de preservativos, HSH, uso de drogas ilícitas, presença de IST, dentre outros^10^.

Neste estudo buscou-se avaliar a prevalência da coinfecção HIV/sífilis em indivíduos vivendo com HIV em início de terapia antirretroviral (TARV) em Belo Horizonte, Minas Gerais, e identificar os fatores associados à coinfecção HIV/sífilis.

## MÉTODOS

Foi realizado um corte seccional de um estudo de coorte prospectivo, denominada Projeto ECOART “Efetividade da terapia antirretroviral em pessoas vivendo com HIV/tuberculose, HIV/hanseníase ou HIV/leishmaniose visceral no Brasil”. Este projeto foi aprovado pelo comitê de ética em pesquisa da Universidade Federal de Minas Gerais (protocolo CAAE 31192914.3.3001.5124, parecer CEP 769.085) e dos serviços participantes. A pesquisa foi realizada de acordo com as instruções da Resolução 466/2012 do Conselho Nacional de Saúde.

A seleção da amostra foi não aleatória, pois todos os indivíduos elegíveis foram convidados a participar do estudo. O recrutamento ocorreu entre setembro de 2015 e outubro de 2017.

O estudo foi realizado em três serviços de assistência especializada (SAE) em HIV/aids do Sistema Único de Saúde (SUS). O serviço I é um ambulatório de assistência de um hospital de referência estadual para o tratamento de doenças infectocontagiosas e dermatologia sanitária; o serviço II é um centro de testagem e aconselhamento (CTA); o serviço III é um SAE referência para o atendimento de doenças infectocontagiosas e parasitárias.

Os critérios de elegibilidade foram: indivíduos de ambos os sexos, com idade igual ou superior a 16 anos, com diagnóstico de HIV independente do tempo de diagnóstico e da condição clínica do indivíduo, em início de TARV (de zero a seis meses de tratamento), sem tratamento farmacológico prévio da infecção por HIV e que estavam em acompanhamento em um dos três serviços selecionados. Todos os participantes concordaram em participar do estudo e assinaram o termo de consentimento livre e esclarecido.

A variável dependente foi o primeiro episódio de sífilis ativa registrado pelo médico em prontuário clínico, em um período de 12 meses após início da TARV.

As variáveis independentes, relacionadas às informações sociodemográficas, comportamentais, clínicas, laboratoriais e ao tratamento farmacológico foram obtidas por meio de entrevistas presenciais, coleta de dados em prontuários clínicos, do Sistema de Controle Logístico de Medicamentos (Siclom) e do Sistema de Controle de Exames Laboratoriais da Rede Nacional de Contagem de Linfócitos CD4+/CD8+ e Carga Viral do HIV (Siscel).

A idade dos indivíduos foi estratificada em faixas etárias para análise descritiva e como variável contínua na regressão logística. A variável cor ou etnia autodeclarada foi estratificada em branca, preta, amarela, parda ou indígena. O estado civil foi dicotomizado em solteiro(a)/divorciado(a)/viúvo(a) ou casado(a)/união estável. A escolaridade foi categorizada em até 9 anos, de 10 a 12 anos e 13 anos ou mais de escolaridade formal para análise descritiva e em duas categorias para regressão logística (até 9 anos, 10 anos ou mais). Investigou-se também a existência de filhos, se tem ou não emprego, renda própria, plano de saúde privado, local de residência e classe econômica.

A variável classe econômica foi avaliada de acordo com critérios brasileiros, como alta (A-B), intermediária (C) e baixa (D-E), nos quais os indivíduos são classificados por meio de grupos socioeconômicos por posse de itens de conforto e nível de escolaridade do chefe familiar. Para análise, a variável foi categorizada em alta (A-B) e intermediária-baixa (C-D-E).

Na avaliação das variáveis comportamentais e hábitos de vida foram analisadas a existência de parceiro sexual fixo em 12 meses após o início da TARV, uso de álcool no mês anterior à entrevista basal, uso de tabaco no momento da entrevista, uso de drogas ilícitas alguma vez na vida (maconha, cocaína, crack e outras, como ecstasy e cola), uso de preservativo no último mês e na última relação sexual.

Foram analisados o tempo médio do diagnóstico do HIV por autorrelato, classificação clínica inicial e final do indivíduo – categorizadas em A (assintomático), B (sintomático) e C (condição clínica definidora de aids), de acordo com os critérios do Centro de Controle e Prevenção de Doenças (CDC) adaptado^13^ –, a presença de comorbidades, presença de outras IST prévias e atuais, presença de lesões em mucosas (oral, genital ou anal), carga viral e contagem de linfócitos TCD4, segundo os dados do Siscel, tempo médio de TARV e esquemas terapêuticos, segundo os dados do Siclom. Os esquemas terapêuticos foram categorizados em tenofovir/lamivudina/efavirenz (TLE), tenofovir/lamivudina/dolutegravir (TLD) e outros (qualquer outro esquema antirretroviral). A não adesão foi avaliada por autorrelato, por meio da pergunta “Você deixou de tomar o medicamento nos últimos 15 dias (sim; não)?”.

A análise descritiva foi realizada por meio de distribuição de frequências para as variáveis categóricas e por meio de medidas de tendência central e de variabilidade para variáveis contínuas. Para a comparação de proporções entre variáveis categóricas utilizou-se o teste qui-quadrado de Pearson ou o exato de Fischer, e para comparação de médias entre variáveis contínuas utilizou-se o teste T. O modelo de regressão logística binária múltipla foi utilizado para avaliar os fatores associados à coinfecção HIV/sífilis.

Foram realizados testes de colinearidade para todas as variáveis. Os resultados da regressão logística foram apresentados por meio de *odds ratio* (OR), intervalo de confiança de 95% (IC95%) e valor p. As variáveis que apresentaram valor p igual ou inferior a 0,20 na análise bivariada foram incluídas no modelo multivariado. Foi utilizado o método *stepwise backward conditional* para obtenção do modelo final. O teste de Hosmer-Lemeshow e a área abaixo da curva *Receiver Operating Characteristics* (ROC) foram utilizados para verificar o ajuste do modelo. As análises estatísticas foram realizadas utilizando os *softwares Statistical Package for the Social Sciences* (SPSS), versão 22.0, e *R Studio*, versão 4.0.2. Todas as análises foram realizadas com nível de significância de 5%.

## RESULTADOS

Foram incluídos no estudo 459 indivíduos (Figura). Dentre as características sociodemográficas da população geral do estudo, observou-se que 81,5% dos indivíduos eram do sexo masculino, com média de idade de 34,7 anos (DP = 10,9), e predominância de faixa etária de 20 a 34 anos (53,4%). A maioria dos indivíduos era solteiro, divorciado ou viúvo (79,7%) e possuía 10 anos ou mais de escolaridade formal (74,3%). Em relação às características comportamentais, 27,5% dos indivíduos faziam uso de tabaco no momento da entrevista, 64,1% faziam uso de álcool em qualquer quantidade no mês anterior à entrevista, 48,1% usaram drogas ilícitas em algum momento na vida. Quanto à potencial fonte de infecção do HIV, mais da metade (51%) era de HSH.

**Figura:** Diagrama dos indivíduos incluídos no estudo.

TARV: terapia antirretroviral.

Dentre os participantes, 25,5% relataram não usar preservativos no último mês e 21,4% na última relação sexual. A classificação clínica A (assintomática) foi observada em 63,4% dos indivíduos na primeira consulta e em 80,8% na última consulta. No início do tratamento, a maioria (88,9%) possuía carga viral detectável e, ao final de 12 meses de TARV, apenas 6,8% apresentou carga viral detectável e o percentual de dados faltantes era de 30,5%. Observou-se, no início da TARV, que 26,1% dos indivíduos apresentaram contagem de linfócitos TCD4 abaixo de 200 células/mm^3^ e 20,9% iniciaram o tratamento com condições clínicas indicadoras de aids. Quanto às características relacionadas ao tratamento, o tempo médio de início da TARV foi de 78,5 dias (DP = 58,97), 63,4% dos indivíduos estavam em uso do esquema TDF/3TC/EFV, 14,8% relataram não adesão ao tratamento e 70,8% tinham acima de três meses de diagnóstico do HIV (Tabela1).

Observou-se uma prevalência de 19,6% (n = 90) de IST registradas no prontuário clínico, sendo que mais da metade dos registros era de sífilis (n = 49). Dentre os 41 indivíduos que tiveram diagnóstico de outras IST, exceto sífilis, observou-se 9,4% (n = 20) de condiloma e verrugas genitais (anal, vaginal, perianal), 5,6% (n = 12) de herpes genital, 5,6% (n = 12) de hepatite B e C, e 1,4% (n = 3) de outras IST (gonorreia/tricomoníase/IST não especificada). Também foi observado registro de outras lesões em mucosa oral, anal ou genital (5%) descritas como lesão peniana, lesão eritematosa em pênis com prurido, lesão anal e lesão genital (dados não mostrados em tabela).

A prevalência de indivíduos coinfectados HIV/sífilis neste estudo foi de 10,6% (n = 49). Quanto às características clínicas desses indivíduos coinfectados, observou-se que a maioria apresentou sífilis não especificada (45%), seguida de sífilis latente e latente tardia (26,6%), secundária (14,3%), terciária (neurossífilis e uveítes) (12,1%) e primária (genital primária) (2%). O tempo médio de registro no prontuário clínico da ocorrência do primeiro episódio da sífilis após início da TARV foi de 115,06 dias (DP = 121,26) e a mediana de 53 dias. O exame informado no registro foi o *Venereal Disease Research Laboratory* (VDRL), com 42,9% de resultados reagentes, e 55,1% não possuíam registro de exames. A Penicilina G Benzatina foi prescrita para 85,7% dos indivíduos em tratamento de sífilis e 4,1% utilizaram outros medicamentos como doxiciclina e ceftriaxona. Observou-se ainda 18,4% (n = 9) de outras IST, além da coinfecção, sendo essas, condiloma e verrugas genitais, hepatite B e C e outras (gonorreia/tricomoníase/IST não especificada). Observou-se ainda, que 10,2% tiveram algum registro de lesões em mucosas oral, anal ou genital (dados não mostrados em tabela).

Conforme demonstrados na Tabela 1, a maioria dos indivíduos coinfectados eram do sexo masculino (93,9%), na faixa etária de 20 a 34 anos (53,1%), pardos ou negros (71,5%), divorciados/solteiros/viúvos (85,7%). Quanto às características comportamentais, a maioria era HSH (69,4%), não possuía parceiro sexual fixo (46,9%) e fazia uso de álcool no mês anterior a entrevista basal (73,5%). Quanto ao uso de preservativo, 26,5% relataram não ter utilizado no último mês e 14,3% não utilizaram na última relação sexual. Houve diferença entre os grupos com e sem coinfecção para as variáveis sexo (p = 0,018), escolaridade formal (p = 0,043), fonte de infecção do HIV (p = 0,025), diagnóstico de outras IST (p = 0,021), tempo médio de diagnóstico do HIV (p = 0,004) e tempo médio de tratamento com antirretrovirais (p = 0,048) (Tabela 1).

**Tabela 1:** Características sociodemográficas, clínicas, comportamentais, hábitos de vida e tratamento das PVHIV, Belo Horizonte, Minas Gerais, 2015–2018 (n = 459).

Características	Total n = 459		Coinfecção HIV/sífilis				p
			Sim n = 49		Não n = 410		
	n	%	n	%	n	%	
**Sociodemográficas**							
Sexo							0,018^a^
Masculino	374	81,5	46	93,9	328	80,0	
Feminino	85	18,5	3	6,1	82	20,0	
Idade (anos)							0,573
16–19	18	3,9	1	2,0	17	4,1	
20–34	245	53,4	26	53,1	219	53,4	
35–49	145	31,6	14	28,6	131	32,0	
≥ 50	51	11,1	8	16,3	43	10,5	
Cor/etnia							0,874^a^
Parda	222	48,4	21	42,9	201	49,0	
Branca	108	23,5	13	26,5	95	23,2	
Preta	106	23,1	14	28,6	92	22,4	
Amarela	15	3,3	1	2,0	14	3,4	
Indígena	3	0,7	0	0,0	3	0,7	
Dados faltantes	5	1,1	0	0,0	5	1,2	
Estado civil							0,271
Solteiro(a)/divorciado(a)/viúvo(a)	366	79,7	42	85,7	324	79,0	
Casado(a)/união estável	93	20,3	7	14,3	86	21,0	
Filhos							0,297
Sim	162	35,3	14	28,6	148	36,1	
Não	297	64,7	35	71,4	262	63,9	
Escolaridade formal (anos)							0,043^a^
≤ 9	117	25,5	11	22,4	106	25,9	
10–12	178	38,8	12	24,5	166	40,5	
≥ 13	163	35,5	26	53,1	137	33,4	
Dados faltantes	1	0,2	0	0,0	1	0,2	
Emprego							0,694
Sim	269	58,6	30	61,2	239	58,3	
Não	190	41,4	19	38,8	171	41,7	
Renda própria							0,087^a^
Sim	373	81,3	41	83,7	332	81,0	
Não	85	18,5	7	14,3	78	19,0	
Dados faltantes	1	0,2	1	2,0	0	0,0	
Classe Econômica							0,306^a^
Alta (A-B)	162	35,3	20	40,8	142	34,6	
Intermediária (C)	212	46,2	23	46,9	189	46,1	
Baixa (D-E)	73	15,9	4	8,2	69	16,8	
Dados faltantes	12	2,6	2	4,1	10	2,4	
Plano privado de saúde							0,511
Sim	121	26,4	11	22,4	110	26,8	
Não	338	73,6	38	77,6	300	73,2	
Local de residência							0,656^a^
Belo Horizonte	404	88,0	44	89,8	360	87,8	
Região metropolitana de Belo Horizonte	49	10,7	4	8,2	45	11,0	
Outros municípios	6	1,3	1	2,0	5	1,2	
**Comportamentais e hábitos de vida**							
Parceiro sexual fixo em 12 meses após a TARV							0,619^a^
Sim	218	47,5	22	44,9	196	47,8	
Não	187	40,7	23	46,9	164	40,0	
Dados faltantes	54	11,8	4	8,2	50	12,2	
Uso de álcool no mês anterior a entrevista							0,435^a^
Sim	294	64,1	36	73,5	258	62,9	
Não	162	35,3	13	26,5	149	36,3	
Dados faltantes	3	0,7	0	0,0	3	0,7	
Uso de tabaco atualmente							0,600
Sim	126	27,5	15	30,6	111	27,1	
Não	333	72,5	34	69,4	299	72,9	
Uso de drogas ilícitas na vida							0,493^a^
Sim	221	48,1	27	55,1	194	47,3	
Não	236	51,4	22	44,9	214	52,2	
Dados faltantes	2	0,4	0	0,0	2	0,5	
Uso de preservativo no último mês							0,156
Sim	248	54,0	31	63,3	217	52,9	
Não	117	25,5	13	26,5	104	25,4	
Dados faltantes	94	20,5	5	10,2	89	21,7	
Uso de preservativo na última relação sexual							0,383^a^
Sim	343	74,7	41	83,7	302	73,7	
Não	98	21,4	7	14,3	91	22,2	
Dados faltantes	18	3,9	1	2,0	17	4,1	
Fonte de infecção do HIV categoria de exposição							0,025^a^
Homens (HSH)	234	51,0	34	69,4	200	48,8	
Homens (não HSH)/mulheres	168	36,6	12	24,5	156	38,0	
Dados faltantes	57	12,4	3	6,1	54	13,2	
**Clínicas**							
Classificação clínica na primeira consulta							0,907^a^
Condições de aids (C)	96	20,9	11	22,4	85	20,7	
Assintomático (A)	291	63,4	30	61,2	261	63,7	
Sintomático (B)	67	14,6	8	16,3	59	14,4	
Dados faltantes	5	1,1	0	0,0	5	1,2	
Classificação clínica na última consulta							0,351^a^
Condições de aids (C)	35	7,6	5	10,2	30	7,3	
Assintomático (A)	371	80,8	42	85,7	329	80,2	
Sintomático (B)	16	3,5	0	0,0	16	3,9	
Dados faltantes	37	8,1	2	4,1	35	8,5	
Comorbidades							0,951^a^
Sim	172	37,5	18	36,7	154	37,6	
Não	279	60,8	31	63,3	248	60,5	
Dados faltantes	8	1,7	0	0,0	8	2,0	
Outras IST (exceto sífilis)							0,021
Sim	41	8,9	9	18,4	32	7,8	
Não	418	91,1	40	81,6	378	92,2	
Lesões em mucosas							0,086^a^
Sim	23	5,0	5	10,2	18	4,4	
Não	436	95,0	44	89,8	392	95,6	
Antecedentes de IST							0,204
Sim	134	29,2	16	32,7	118	28,8	
Não	124	27,0	9	18,4	115	28,0	
Dados faltantes	201	43,8	24	49,0	177	43,2	
**Laboratoriais**							
Carga viral no início da TARV (cópias/ml)							0,812^a^
≤ 100 mil	305	66,4	31	63,3	274	66,8	
> 100 mil	114	24,8	14	28,6	100	24,4	
Dados faltantes	40	8,7	4	8,2	36	8,8	
Carga viral no início da TARV							0,194^a^
Indetectável	11	2,4	3	6,1	8	2,0	
Detectável	408	88,9	42	85,7	366	89,3	
Dados faltantes	40	8,7	4	8,2	36	8,8	
Carga viral após 12 meses de TARV (cópias/ml)							0,211^a^
≤ 100 mil	316	68,8	39	79,6	277	67,6	
> 100 mil	3	0,7	0	0,0	3	0,7	
Dados faltantes	140	30,5	10	20,4	130	31,7	
Carga viral após 12 meses de TARV							0,203
Indetectável	288	62,7	34	69,4	254	62,0	
Detectável	31	6,8	5	10,2	26	6,3	
Dados faltantes	140	30,5	10	20,4	130	31,7	
Linfócitos TCD4 no início de TARV (células/mm^3^)							0,274^a^
< 200	120	26,1	12	24,5	108	26,3	
200–500	165	35,9	13	26,5	152	37,1	
> 500	134	29,2	20	40,8	114	27,8	
Dados faltantes	40	8,7	4	8,2	36	8,8	
Linfócitos TCD4 após 12 meses de TARV (células/mm^3^)							0,998
< 200	27	5,9	3	6,1	24	5,9	
200–500	139	30,3	15	30,6	124	30,2	
> 500	155	33,8	16	32,7	139	33,9	
Dados faltantes	138	30,1	15	30,6	123	30,0	
**Tratamento farmacológico**							
Tempo médio do diagnóstico de HIV (mês)	15,3	DP = 32,1	8,5	DP = 13,4	16,1	DP = 33,5	0,004^b^
Tempo do diagnóstico de HIV (mês)							0,018^a^
≤ 3	131	28,5	21	42,9	110	26,8	
> 3	325	70,8	27	55,1	298	72,7	
Dados faltantes	3	0,7	1	2,0	2	0,5	
Tempo médio de tratamento com TARV (dias)	78,6	DP = 59,0	62,8	DP = 55,2	80,4	DP = 59,2	0,048^b^
Esquema terapêutico em uso							0,085^a^
TLE	291	63,4	38	77,6	253	61,7	
TLD	142	30,9	9	18,4	133	32,4	
Outros esquemas	26	5,7	2	4,1	24	5,9	
Não adesão à TARV nos últimos 15 dias							0,339
Sim	68	14,8	9	18,4	59	14,4	
Não	362	78,9	35	71,4	327	79,8	
Dados faltantes	29	6,3	5	10,2	24	5,9	

PVHIV: pessoas vivendo com HIV; HSH: homens que fazem sexo com homens; IST: infecções sexualmente transmissíveis; TLE: tenofovir/lamivudina/efavirenz; TLD: tenofovir/lamivudina/dolutegravir; TARV: terapia antirretroviral; DP: desvio padrão.

^a^ Teste exato de Fischer.

^b^ Teste T para comparação de médias.

As características que foram significativamente associadas a maior chance de ter a coinfecção HIV/sífilis, na análise bivariada, foram sexo masculino, ser HSH e ter diagnóstico de outras IST. As características associadas a menor chance de ter a coinfecção HIV/sífilis foram maior tempo de TARV e uso do esquema antirretroviral TLD (Tabela 2).

**Tabela 2:** Análise bivariada dos fatores associados à coinfecção HIV/sífilis, Belo Horizonte, Minas Gerais, 2015–2018 (n = 459)

Características	n (%)^a^	OR (IC95%)	p
**Sociodemográficas**			
Sexo			
Masculino	374 (81,5)	3,83 (1,16–12,64)	0,027
Feminino	85 (18,5)	1,00	
Idade (anos)	459 (100)	1,01 (0,98–1,03)	0,674
Cor/etnia			
Parda/preta	328 (72,2)	0,96 (0,50–1,84)	0,892
Branca/amarela/indígena	126 (27,8)	1,00	
Estado civil			
Solteiro(a)/divorciado(a)/viúvo(a)	366 (79,7)	1,59 (0,69–3,67)	0,275
Casado(a)/união estável	93 (20,3)	1,00	
Filhos			
Sim	162 (35,3)	1,00	
Não	297 (64,7)	1,41 (0,74–2,71)	0,299
Escolaridade formal (anos)			
≤ 9	117 (25,5)	0,83 (0,41–1,68)	0,599
≥ 10	341 (74,5)	1,00	
Emprego			
Sim	269 (58,6)	1,00	
Não	190 (41,4)	0,89 (0,48–1,63)	0,694
Renda própria			
Sim	373 (81,4)	1,00	
Não	85 (18,6)	0,73 (0,31–1,68)	0,456
Classe econômica			
Alta (A-B)	162 (36,2)	1,00	
Intermediária-baixa (C-D-E)	285 (63,8)	0,74 (0,40–1,37)	0,343
Plano privado de saúde			
Sim	121 (26,4)	1,00	
Não	338 (73,6)	1,27 (0,63–2,57)	0,511
Local de residência			
Região metropolitana/outros municípios	55 (12,0)	1,00	
Belo Horizonte	404 (88,0)	1,22 (0,46–3,23)	0,685
**Comportamentais e hábitos de vida**			
Parceiro sexual fixo em 12 meses após a TARV			
Sim	218 (47,5)	1,00	
Não	187 (40,7)	1,25 (0,67–2,32)	0,482
Uso de álcool no mês anterior a entrevista			
Sim	294 (64,5)	1,60 (0,82–3,11)	0,167
Não	162 (35,5)	1,00	
Uso de tabaco atualmente			
Sim	126 (27,5)	1,19 (0,62–2,27)	0,600
Não	333 (72,5)	1,00	
Uso de droga ilícita na vida			
Sim	221 (48,4)	1,35 (0,75–2,46)	0,319
Não	236 (51,6)	1,00	
Uso de preservativo no último mês			
Sim	248 (67,9)	1,00	
Não	117 (32,1)	0,88 (0,44–1,74)	0,704
Uso de preservativo na última relação sexual			
Sim	343 (77,8)	1,00	
Não	98 (22,2)	0,57 (0,25–1,31)	0,182
Fonte de infecção do HIV – categoria de exposição			
Homens (HSH)	234 (51,0)	2,21 (1,11–4,41)	0,024
Homens (não HSH)/mulheres	168 (36,6)	1,00	
**Clínicas**			
Classificação clínica na primeira consulta			
Condições de aids (C)	96 (21,1)	1,00	
Sem aids (A-B)	358 (78,9)	1,09 (0,54–2,22)	0,813
Classificação clínica na última consulta			
Condições de aids (C)	35 (8,3)	1,00	
Sem aids (A-B)	387 (91,7)	0,73 (0,27–1,98)	0,538
Comorbidades			
Sim	172 (38,1)	0,94 (0,51–1,73)	0,830
Não	279 (61,9)	1,00	
Outras IST (exceto sífilis)			
Sim	41 (8,9)	2,66 (1,19–5,96)	0,018
Não	418 (91,1)	1,00	
Lesões em mucosas			
Sim	23 (5,0)	2,48 (0,88–6,99)	0,087
Não	436 (95,0)	1,00	
**Laboratoriais**			
Carga viral no início de TARV			
Detectável	408 (88,9)	1,00	
Indetectável	11 (2,4)	3,27 (0,84–12,79)	0,089
Carga viral após 12 meses de TARV			
Detectável	31 (6,8)	1,00	
Indetectável	288 (62,7)	0,70 (0,25–1,93)	0,487
Linfócito TCD4 no início de TARV (células/mm^3^)			
< 200	120 (26,1)	1,00	
200 a 500	165 (35,9)	0,77 (0,34–1,75)	0,533
> 500	134 (29,2)	1,58 (0,74–3,39)	0,240
Linfócito TCD4 após 12 meses de TARV (células/mm^3^)			
< 200	27 (5,9)	1,00	
200 a 500	139 (30,3)	0,97 (0,26–3,60)	0,961
> 500	155 (33,8)	0,92 (0,25–3,40)	0,902
**Tratamento farmacológico**			
Tempo diagnóstico HIV (meses)	456 (100,0)	0,99 (0,97–1,01)	0,138
Tempo de tratamento com antirretrovirais (dias)	459 (100,0)	0,99 (0,99–1,00)	0,050
Esquema terapêutico em uso			
TLE	291 (63,4)	1,00	
TLD	142 (30,9)	0,45 (0,21–0,96)	0,039
Outros esquemas	26 (5,7)	0,56 (0,13–2,44)	0,436
Autorrelato de não adesão à TARV nos últimos 15 dias			
Sim	68 (15,8)	1,43 (0,65–3,12)	0,375
Não	362 (84,2)	1,00	

OR: *odds ratio*; IC95%: intervalo de confiança de 95%; TARV: terapia antirretroviral; HSH: homens que fazem sexo com homens; IST: infecções sexualmente transmissíveis; TLE: Tenofovir/Lamivudina/Efavirenz; TLD: Tenofovir/Lamivudina/Dolutegravir.

^a^ Os números variam conforme os dados ignorados.

Na análise multivariada (Tabela 3) as características independentes associadas à maior chance de coinfecção foram ter diagnóstico de outras IST (OR = 3,33; IC95% 1,24–8,95) e uso de álcool no mês anterior a entrevista (OR = 2,30; IC95% 1,01–5,26). As variáveis sexo e tempo de tratamento com antirretrovirais permaneceram no modelo final, porém não apresentaram significância estatística.

**Tabela 3:** Regressão logística múltipla dos fatores associados à coinfecção HIV/sífilis, Belo Horizonte, Minas Gerais, 2015–2018 (n = 349a).

Características	OR (IC95%)	p
Sexo		
Masculino	3,58 (0,82–15,58)	0,089
Feminino	1,00	
Uso de álcool no mês anterior a entrevista		
Sim	2,30 (1,01–5,26)	0,049
Não	1,00	
Diagnostico de outras IST (exceto sífilis)		
Sim	3,33 (1,24–8,95)	0,017
Não	1,00	
Tempo de tratamento com antirretrovirais (dias)	0,99 (0,99–1,00)	0,066

OR: *odds ratio*; IC95%: intervalo de confiança de 95%. IST: infecções sexualmente transmissíveis; ROC: *Receiver Operating Characteristics*.

^a^ 110 pacientes com dados faltantes em covariáveis foram excluídos do modelo final.

Ajuste do modelo: teste de Hosmer e Lemeshow: X2 = 7,66; df = 8; valor-p = 0,468; área sob a curva ROC = 0,688.

## DISCUSSÃO

As pessoas vivendo com HIV (PVHIV) atendidas em três serviços de assistência especializada em HIV no SUS, em Belo Horizonte, que estavam em início de TARV apresentaram alta prevalência de coinfecção por IST (19,6%), sendo a sífilis (10,6%) a mais prevalente. Destaca-se que a prevalência global estimada de sífilis entre homens e mulheres, sem a infecção por HIV, é de 0,5% no Brasil^1^. As características associadas independentemente com a coinfecção HIV/sífilis foram diagnóstico de outras IST e uso de álcool no mês anterior à entrevista.

A prevalência e os fatores associados à sífilis nas PVHIV apresentam variações, tanto em estudos brasileiros quanto internacionais. Essa variação depende do tipo de população, como por exemplo a população chave (transgêneros, profissionais do sexo, pessoas que usam drogas injetáveis, HSH e prisioneiros – e seus parceiros) que têm prevalência maior de coinfecção HIV/sífilis^14^.

A prevalência de coinfecção HIV/sífilis observada neste estudo foi menor que aquela encontrada em um estudo realizado em Mkushi, na Zâmbia, no qual os autores observaram 40,5% de coinfecção HIV/sífilis em indivíduos recém-diagnosticados com HIV iniciando TARV^15^. Também foi menor do que a prevalência encontrada em um estudo prospectivo multicêntrico com HSH, realizado na Alemanha, que foi de 39,6%^16^. Em outro estudo realizado no Brasil, com trabalhadores do sexo, a prevalência encontrada foi de 30,8%^17^.

Semelhante a outros estudos^18,19^, também observamos que ter diagnóstico de outras IST apresentaram associação independente com maior chance de coinfecção HIV/sífilis. Esse resultado pode indicar que o comportamento sexual de risco das PVHIV pode contribuir para a disseminação da infecção pelo HIV e impactar no controle da sua transmissão.

As IST são transmitidas por contato sexual sem o uso de preservativo, uma medida preventiva importante entre casais HIV sorodiscordantes e soroconcordantes para evitar a transmissão de outras IST. Um estudo encontrou a presença de sífilis, citomegalovírus, papilomavírus humano (HPV) e herpes simples nos HSH que vivem com HIV^20^. Ainda, de forma semelhante em nosso estudo, foi observada a presença das IST condiloma e verrugas genitais, herpes genital, hepatite B e C e outras (gonorreia/tricomoniase/IST não especificada), além de lesões em mucosas anal e genital.

As PVHIV apresentam um risco maior de coinfecção com hepatites e sífilis do que a população em geral. As infecções bacterianas, os protozoários, a herpes genital e infecções sexuais anteriores, foram descritas como fatores de risco para a coinfecção HIV/sífilis. As IST podem apontar um comportamento sexual de risco das PVHIV, elevar a possibilidade de infecção do HIV e impactar no controle da sua transmissão^20^_._

Em outro estudo foram analisados os fatores associados à coinfecção HIV/IST em 295 PVHIV, em que 37% tiveram ao menos uma IST. Dentre as IST citadas, 32% delas eram sífilis, 16% de gonorreia e 8% de clamídia. A alta prevalência de IST entre PVHIV sugere a necessidade de testagem, prevenção e tratamento adequado nessa população^21^.

Neste estudo, o relato de uso de álcool anterior a entrevista apresentou associação com maior chance de coinfecção HIV/sífilis, resultado semelhante a outros estudos^22,23^. O consumo de álcool é um grave problema de saúde pública, visto que pode levar o indivíduo à adoção de práticas sexuais de risco e contribuir para a carência de hábitos preventivos às IST, como não usar preservativo, trocar de parceiros com frequência e praticar sexo em grupo ou anal, acarretando maiores chances de contrair sífilis e outras IST. Esse cenário colabora para a manutenção da cadeia de transmissão das IST na PVHIV^24^_._

O álcool é uma substância que deprime o sistema nervoso central e atua reduzindo a ansiedade e a inibição. A convicção de que usar álcool aumenta o prazer, faz com que seja utilizado antes ou durante as práticas sexuais. Estima-se que o consumo de álcool entre PVHIV é 2,5 vezes maior do que no restante da população. O uso de álcool e drogas aumenta em até seis vezes o risco de pessoas com HIV praticarem sexo sem preservativo e de terem múltiplos parceiros. Em um estudo foi estimado a prevalência de 28,6% de uso abusivo de álcool entre pessoas que vivem com HIV^25^_._

Maior tempo de TARV apresentou associação à menor chance de coinfecção HIV/sífilis e permaneceu no modelo final devido a maior robustez, embora não tenha apresentado significância estatística. Cabe ressaltar que são controversos os resultados dos estudos nos quais avaliou-se a associação entre uso de TARV e transmissão de IST.

Em um estudo de coorte retrospectiva encontrou-se associação do uso de TARV com menor chance de coinfecção HIV/sífilis no início do tratamento, corroborando com o resultado encontrado nesta pesquisa. Em contrapartida, o uso da TARV foi associado a maior chance de coinfecção nos indivíduos que tiveram soroconversão de sífilis durante o acompanhamento desses indivíduos^26^. No estudo de Tsachouridou et al.^27^ os indivíduos em uso de TARV apresentaram 2,4 vezes mais chance de ter coinfecção HIV/sífilis. Esses estudos apontam que as vantagens do uso dos antirretrovirais refletem no comportamento sexual das PVHIV ao não usarem o preservativo por se sentirem, provavelmente, seguros quanto a não transmissão do vírus HIV^27^.

Em nosso estudo, a variável sexo masculino apresentou associação para maior chance de coinfecção HIV/sífilis e permaneceu no modelo final devido à robustez do modelo, embora não tenha apresentado significância estatística. Esse resultado foi consonante ao de outros estudos que apontaram maior risco de coinfecção em pessoas do sexo masculino^28,29^.

A prevalência da sífilis e as distintas IST encontradas neste estudo podem refletir o uso inconsistente do preservativo e de outras ações de prevenção dessas infecções. O conhecimento sobre os fatores associados à prevalência da coinfecção HIV/sífilis pode subsidiar a tomada de decisões dos profissionais inseridos no cuidado às PVHIV. O acompanhamento e tratamento adequado da sífilis e IST requerem orientações de práticas sexuais seguras para prevenção dessas coinfecções nas PVHIV.

As limitações do estudo dizem respeito à utilização de dados secundários com elementos faltantes de registros gerais sobre as informações clínicas e sobre os exames laboratoriais dos indivíduos.

Este estudo tem como pontos fortes a qualidade e tratamento dos dados primários coletados, com análise de confiabilidade de 10% da amostra total para coleta e digitação. Ressalte-se a alta concordância interdigitador perfeita avaliada pela estatística Kappa, a inclusão abrangente de variáveis explicativas e a robustez do modelo final.

Conclui-se que a prevalência de IST registradas foi alta e a sífilis foi a coinfecção mais prevalente. O uso de álcool e o diagnóstico de outras IST foram associadas a maior chance de coinfecção HIV/sífilis nessa população.
